# A risk-model for hospital mortality among patients with severe sepsis or septic shock based on German national administrative claims data

**DOI:** 10.1371/journal.pone.0194371

**Published:** 2018-03-20

**Authors:** Daniel Schwarzkopf, Carolin Fleischmann-Struzek, Hendrik Rüddel, Konrad Reinhart, Daniel O. Thomas-Rüddel

**Affiliations:** 1 Integrated Research and Treatment Center–Center for Sepsis Control and Care, Jena University Hospital, Jena, Germany; 2 Department of Anesthesiology and Intensive Care Medicine, Jena University Hospital, Jena, Germany; Azienda Ospedaliero Universitaria Careggi, ITALY

## Abstract

**Background:**

Sepsis is a major cause of preventable deaths in hospitals. Feasible and valid methods for comparing quality of sepsis care between hospitals are needed. The aim of this study was to develop a risk-adjustment model suitable for comparing sepsis-related mortality between German hospitals.

**Methods:**

We developed a risk-model using national German claims data. Since these data are available with a time-lag of 1.5 years only, the stability of the model across time was investigated. The model was derived from inpatient cases with severe sepsis or septic shock treated in 2013 using logistic regression with backward selection and generalized estimating equations to correct for clustering. It was validated among cases treated in 2015. Finally, the model development was repeated in 2015. To investigate secular changes, the risk-adjusted trajectory of mortality across the years 2010–2015 was analyzed.

**Results:**

The 2013 deviation sample consisted of 113,750 cases; the 2015 validation sample consisted of 134,851 cases. The model developed in 2013 showed good validity regarding discrimination (*AUC* = 0.74), calibration (observed mortality in 1^st^ and 10^th^ risk-decile: 11%-78%), and fit (*R*^*2*^ = 0.16). Validity remained stable when the model was applied to 2015 (AUC = 0.74, 1^st^ and 10^th^ risk-decile: 10%-77%, *R*^*2*^ = 0.17). There was no indication of overfitting of the model. The final model developed in year 2015 contained 40 risk-factors. Between 2010 and 2015 hospital mortality in sepsis decreased from 48% to 42%. Adjusted for risk-factors the trajectory of decrease was still significant.

**Conclusions:**

The risk-model shows good predictive validity and stability across time. The model is suitable to be used as an external algorithm for comparing risk-adjusted sepsis mortality among German hospitals or regions based on administrative claims data, but secular changes need to be taken into account when interpreting risk-adjusted mortality.

## Introduction

Sepsis is the major cause of death from infectious diseases [1]. Most of these deaths are considered to be preventable [2–4] and numerous quality improvement initiatives demonstrated that sepsis-related mortality can be considerably reduced [5–7]. Recognizing existing severe shortcomings in prevention and treatment of sepsis, a WHO resolution released in May 2017 urged the member states to develop national policies to improve sepsis care [4].

There is consensus that valid measurement of provider performance is central to improve quality of care [8]. Obtaining prospective clinical data for performance measurement is costly and possibly prevents hospitals from participating in quality improvement [9, 10]. Administrative or claims data are readily available, include all patients diagnosed with the condition of interest, and are widely used for public reporting of hospital performance [11, 12]. Administrative claims are already used to obtain inpatient quality indicators–among them sepsis-related mortality–within a voluntary quality collaborative involving more than 300 German hospitals [13].

Differences in case-mix between hospitals might bias comparisons of raw mortality rates. Thus, statistical risk-adjustment needs to be applied [14, 15]. Since the existing German voluntary sepsis quality indicator is only adjusted for age and gender, the validity of hospital comparisons is questionable. An US-American risk-model for inpatient sepsis mortality solely based on claims data has recently been developed [16], but as such risk-models are not valid if used outside the population they were designed for, a similar model needs to be developed for Germany. The aim of this study was therefore to develop a risk-model suitable for risk-adjusted comparison of sepsis-related mortality between German hospitals that is solely based on administrative claims data.

## Materials and methods

The Agency for Health Care Research and Quality Improvement (AHRQ) of the US Department for Health and Human Services provides hospitals with a methodology for claims-based measurement and risk-adjusted comparison of mortality in several conditions [17]. Risk-models are developed based on the Nationwide Inpatient Sample [18] and can be used as an external risk adjustment algorithm by US hospitals to compare their performance against the national standard [19, 20]. We adopted this methodology to develop a risk-model on sepsis-related mortality using German national claims data [21].

### Data source

This study uses German diagnosis related groups (DRG) data of patients treated between 2010 and 2015 [21]. By federal law on reimbursement of health care providers (§21 Krankenhausentgeltgesetz [KhEntgG]), all hospitals are obliged to annually provide their DRG data to a federal administrative body. With a time-lag of 1.5 years the data of all cases are made available for scientific analysis in anonymized form by the Federal Statistical Office (Deutsches Statistisches Bundesamt) via remote computing. German DRG data contain information on patient demographics, primary and secondary diagnoses (ICD-10-GM: International Classification of Diseases and Related Health Problems- German Modification), procedures and general medical measures (OPS: Operationen und Prozedurenschlüssel [German Procedure Classification]), treating departments, hospital length of stay, type of admission, and type of discharge. ICD-10 codes have no time-flag, OPS codes have a time-flag but this mostly indicates rather the time of coding than the time the procedure was performed. Hospitals are identified in the DRG data by a unique institutional identifier (IK: Institutionskennzeichen). More than one hospital site of the same institution might use the same IK, but typically these sites are organizationally linked and mutually dependent.

### Study population

The target population were cases with severe sepsis or septic shock according to modified American College of Chest Physicians/Society of Critical Care Medicine criteria [22], identified by presence of the respective clinical ICD-10 codes (severe sepsis: R65.1, septic shock: R57.2), and of at least 15 years of age. We focused on severe sepsis and septic shock since sepsis without organ dysfunction is excluded from recent Sepsis-3 consensus definitions [1].

#### Derivation and validation cohorts

Because of the time-lag of 1.5 years for availability of national DRG data, the validity of a risk-model for predicting mortality in cases treated two years after its development needed to be proven. Therefore, a risk-model was developed based on cases treated in 2013 (derivation cohort) and tested for its validity in 2014 and 2015 (validation cohorts). The final model was then developed based on cases treated in 2015. Additionally, secular trends in sepsis-related mortality were investigated using cases treated between 2010 and 2015.

### Outcome

The outcome was all-cause hospital mortality among patients with severe sepsis or septic shock. Hospital mortality could not be corrected for transfer to other hospitals since anonymized data did not allow identification of individual patients with several linked hospital stays. Cases with admission by transfer from another hospital and cases with discharge to another hospital were not excluded from the analysis.

### Model derivation

#### Predictors of mortality

Based on clinical reasoning and existing research, candidate variables were chosen from patient demographics [16, 23], hospital admission type [24], clinical characteristics of the infection [23–25], comorbidities [16, 23], and specific procedures.

1. *Patient demographics* included gender and age. To allow for non-linear effects of age, the quadratic and cubic polynomials were included. Age was transformed by mean-centering and standardization to decades (age_t_ = [age– 70])/10).
2. *Hospital admission* type was categorized in ‘emergency admission’, ‘referral by physician or dentist’, ‘transfer from another hospital with length of stay ≥ 24h’, and ‘transfer from rehabilitation clinic or another hospital with length of stay <24h’. Additionally, an indicator variable for admission by a surgical department was defined based on standardized codes for medical specialty of a department to identify the subgroup of surgical patients.
3. *Clinical characteristics of the infection* included focus of infection defined by presence of specific ICD-10 codes, and presence of an explicit sepsis code as a primary diagnosis. Focus of infection codes were chosen based on existing international literature [26–28] and clinical knowledge. Two junior physicians independently defined indicator variables for specific foci and consensus was created in discussions with two experienced intensivists. A primary diagnosis of sepsis was defined if an explicit sepsis code (A40.–A41., R57.2) was present as primary diagnosis. Finally, infection by multi-resistant pathogens was defined by presence of an OPS-code for treatment of multiresistant pathogens.
4. *Comorbidities* were defined by the categories of the Charlson Comorbidity Index (CCI) and the Elixhauser Comorbidity Index (ECI) [29, 30] based on a German adaptation of a previously developed ICD-10 coding algorithm [31, 32]. Duplicate CCI and ECI categories were excluded. Additional categories for leukemia, congenital and acquired asplenia were introduced.
5. *Specific procedures* defined by respective OPS codes were used to identify patient subgroups with especially high risk of dying: patients receiving palliative care, treatment of stroke, and chemotherapy.

Univariate relations between predictors were investigated, to avoid problems of multicollinearity. Highly correlated predictors were collapsed or one predictor was excluded based on clinical judgment and univariate relations to hospital mortality. Only conditions with incidence of at least 1% were considered as candidate variables [33, 34]. S1 Table presents the definitions of candidate variables.

#### Model development

Risk factors were first selected from the set of candidate variables by a standard logistic regression model with backward elimination applied to the 2013 derivation cohort. Since patients with septic shock are a distinctive subgroup with higher mortality, we wanted to make the model suitable also for comparing hospital mortality within the subgroups of cases with or without septic shock. Risk-factors might have different effects within these respective subgroups, which can be modeled by statistical interaction effects. Therefore, interaction effects of the selected predictors with the presence of a diagnosis of septic shock were also included and backward-selected in a second modeling step. This was done to increase the validity of the model when using it within the subgroups of patients without septic shock or with septic shock. Given the large sample size, the exclusion criterion was set to *p* > 0.0003 based on a power-analysis (S1 Appendix). Resembling the approach of the AHRQ [20], the selected risk-model was refitted by a logistic model using generalized estimating equations (GEE) with an exchangeable covariance matrix to control for clustering of cases into hospitals [35]. After validating the 2013 risk-model, the full model development process was repeated based on cases treated in 2015 to obtain a model suitable for provider comparison based on most-recent data.

### Model validation

#### Predictive performance of the model

To evaluate the predictive performance of the model identified in the derivation cohort (“derivation model”), the area under the curve (AUC) was used as measure of discrimination, the distribution of observed mortality across the deciles of predicted risk was used as a measure of calibration, and the squared Pearson correlation (R2) between hospital mortality and the log-odds of predicted mortality was used as measure of explained variation [36].

#### Stability of the model

Because of the 1.5 years time-lag in availability of national DRG data, the stability of the risk-models over time had to be tested. The validity of the derivation model was assessed in the 2014 and 2015 validation cohorts, respectively. This was done by using the identified risk-factors and coefficients estimates to predict mortality risk and log-odds of mortality risk among the cases of the validation cohorts. These predicted values were used to calculate AUC, distribution of observed mortality across deciles of risk, and R2 within the validation cohorts. Additionally, overfitting indices were calculated to assess if too many risk-factors were included in the models [33]. For this purpose, predicted log-odds of mortality risk were introduced as sole predictor in logistic regression models using GEE within each validation cohort. Coefficients estimates of intercepts and slopes were used to assess overfitting. Because of the known secular trend of decreasing sepsis mortality [37–40], the intercepts were expected to deviate from the ideal value of 0. A substantial deviation of the slope from its ideal value of 1 would be an indicator of overfitting. Analyses were conducted using SAS® software, version 9.2 (SAS Institute, Cary NC).

## Results

Table 1 provides an overview of the study samples between 2010 and 2015. Incidence of severe sepsis/septic shock increased from 0.5% to 0.73% of all hospital episodes. After exclusion of cases with age <15 years, 113,750 cases in the derivation cohort of 2013 and 134,851 cases in the validation cohort of 2015 remained for analysis (S1 Fig). Proportion of cases with septic shock increased from 25.6% in 2010 to 30.2% in 2015. Hospital mortality among cases with severe sepsis or septic shock decreased from 48.4% to 42%.

**Table 1:** Sample of cases with severe sepsis or septic shock in German national diagnostic related groups data.

	2010	2011	2012	2013	2014	2015
**Cases with severe sepsis or septic shock**^**a**^						
*n (%* of total hospitalizations)	87,990 (0.5%)	96,564 (0.55%)	105,147 (0.58%)	115,426 (0.64%)	123,296 (0.67%)	136,545 (0.73%)
**Exclusion criteria**						
Age < 15 years						
*n* (*%* of cases with severe sepsis or septic shock)	1,560 (1.77%)	1,665 (1.72%)	1,584 (1.51%)	1,676 (1.45%)	1,513 (1.23%)	1,694 (1.24%)
Final analysis sample						
*n* (*%* of cases with severe sepsis or septic shock)	86,430 (98.23%)	94,899 (98.23%)	103,563 (98.49%)	113,750 (98.55%)	121,783 (98.77%)	134,851 (98.76%)
Hospital mortality: *n* (*%*)	41,813 (48.38%)	44,198 (46.57%)	46,418 (44.82%)	50,073 (44.02%)	51,313 (42.13%)	56,579 (41,96%)
Cases with severe sepsis without shock						
*n* (*%* of final sample)	64,314 (74.41%)	67,993 (71.65%)	73,111 (70.6%)	80,184 (70.49%)	85,116 (69.89%)	94,189 (69.85%)
Hospital mortality: *n* (*%*)	28,267 (43.95%)	28149 (41.4%)	28,468 (38.94%)	30,260 (37.74%)	30,413 (35.73%)	33,216 (35.27%)
Cases with septic shock						
*n* (*%* of final sample)	22,116 (25.59%)	26,906 (28.35%)	30,452 (29.4%)	33,566 (29.51%)	36,667 (30.11%)	40,662 (30.15%)
Hospital mortality: *n* (*%*)	13,546 (61.25%)	16,049 (59.65%)	17,950 (58.95%)	19,813 (59.03%)	20,900 (57%)	23,363 (57.46%)

^a^ Cases with severe sepsis defined by presence of ICD-10 code R65.1; cases with septic shock defined by presence of ICD-10 code R57.2.

**Fig 1:**
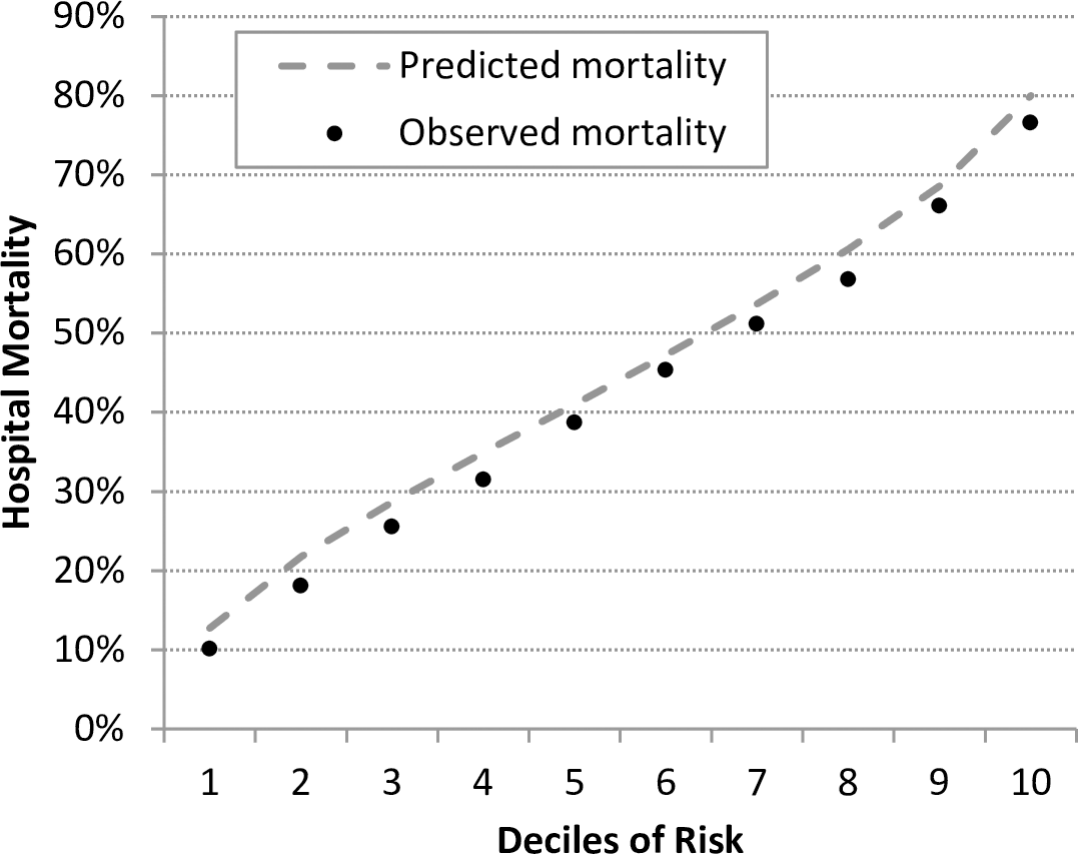
Calibration of the model derived in 2013 for predicting hospital mortality in cases with severe sepsis or septic shock treated in 2015. The gap between predicted and observed mortality (M±SD = 2.7%±0.7%) results from a secular trend with decreasing mortality (compare Fig 2).

After univariate analyses, clinical review of candidate variables and backward selection in the 2013 derivation cohort, 42 risk-factors and 13 interaction effects with septic shock were identified (S2 Table). The model had good discrimination (*AUC* = 0.737; 95% *CI*: 0.734, 0.74; *p* < 0.001), calibration (mortality rate in lowest risk-decile and highest decile: 0.11–0.78) and fit (*R*^*2*^ = 0.16). Concerning stability, the model showed similar validity statistics in the two validation cohorts of 2014 and 2015 (Table 2). There was no indication of overfitting of the model. As expected based on known secular trends, the intercepts deviated from 0, but the slopes were close to 1. A secular trend is also apparent in the calibration plot (Fig 1), where mortality observed in 2015 is slightly lower than mortality predicted from the 2013 model. Beside this, the 2013 model was well calibrated across the deciles of risk also when used on data of 2015.

**Table 2:** Validity of logistic regression model fitted by generalized estimation equations for hospital mortality in cases with severe sepsis or septic shock in derivation and validation samples.

		Validity of model			
Samples	*n*	Overfitting indices (Intercept, Slope)	*R*^*2*^	Calibration, Lowest decile-Highest decile	*AUC* (95*% CI*)
Derivation sample					
2013	113,750	(0, 1)	0.16	0.11–0.78	0.737 (0.734, 0.74)
Validation samples					
2014	121,783	(-0.08, 0.98)	0.16	0.1–0.76	0.736 (0.733, 0.739)
2015	134,851	(-0.08, 1)	0.17	0.1–0.77	0.739 (0.737, 0.743)

Predictors selected by backward elimination in a logistic regression model. Model was refit using generalized estimation equations with an exchangeable correlation matrix to control for clustering of cases in hospitals. *R*^*2*^: Estimation of explained variance by the squared Pearson correlation of predicted values with the observed hospital mortality (0: alive, 1: deceased); *AUC*: Area under the curve.

The model development was repeated for cases treated in 2015, identifying 40 risk-factors and 11 interaction effects with diagnosis of septic shock. Table 3 shows the risk-factors with coefficients estimates and odds ratios. Selected risk-factors and coefficient estimates closely resemble the model selected in 2013 (S2 Table).

**Table 3:** Coefficients estimates of logistic regression model using generalized estimation equations for hospital mortality in cases with severe sepsis or septic shock treated in 2015.

Variables	*Mean±SD* or *%*	Estimate	SE	P-value	Odds Ratio	95*%* CI
Intercept		-0.38	0.03	<0.001		
Female gender	41.24%	0.14	0.01	<0.001	1.16	1.13–1.18
Age (estimate based on transformed value)^a^	70.86***±***13.93	0.4	0.01	<0.001	1.49	1.47–1.52
Age^2^		0.04	0	<0.001	1.04	1.04–1.05
Age^3^		0.01	0	<0.001	1.01	1–1.01
Reason for admission: Emergency (reference)	66.81%					
Referral by physician or dentist	21.17%	-0.13	0.02	<0.001	0.88	0.85–0.91
Hospital transfer with pre-treatment >24h	9.32%	0.22	0.02	<0.001	1.24	1.19–1.3
Hospital transfer with pre-treatment < 24h or rehabilitation hospital	2.69%	-0.1	0.04	0.021	0.91	0.84–0.99
Septic shock	30.15%	1.09	0.03	<0.001	2.98	2.79–3.19
Infection of central nervous system	1.46%	-0.22	0.06	<0.001	0.8	0.72–0.89
Foreign body associated infection	9.85%	-0.33	0.02	<0.001	0.72	0.69–0.76
Infection of vascular system	4.69%	-0.31	0.03	<0.001	0.73	0.69–0.78
Infection of upper respiratory tract	1.49%	-0.15	0.05	0.001	0.86	0.78–0.94
Soft tissue and wound infections	6.93%	-0.29	0.03	<0.001	0.75	0.71–0.78
CCI: Cerebrovascular disease	13.02%	0.19	0.02	<0.001	1.21	1.16–1.28
CCI: Myocardial infarction	9.91%	0.11	0.02	<0.001	1.12	1.08–1.17
CCI: Mild liver disease	7.85%	0.21	0.03	<0.001	1.24	1.17–1.31
CCI: Moderate or severe liver disease	3.12%	0.86	0.04	<0.001	2.37	2.19–2.56
ECI: Blood loss anemia	1.11%	-0.27	0.06	<0.001	0.76	0.67–0.86
ECI: Cardiac arrhythmias	43.21%	0.07	0.01	<0.001	1.08	1.05–1.11
ECI: Congestive heart failure	36.33%	0.18	0.02	<0.001	1.19	1.16–1.23
ECI: Coagulopathy	32.13%	0.37	0.02	<0.001	1.44	1.4–1.49
ECI: Deficiency anemia	4.93%	-0.38	0.03	<0.001	0.68	0.64–0.73
ECI: Depression	6.29%	-0.45	0.03	<0.001	0.64	0.6–0.67
ECI: Drug abuse	1.34%	-0.38	0.07	<0.001	0.69	0.6–0.79
ECI: Hypertension, complicated	11.33%	-0.54	0.03	<0.001	0.58	0.55–0.61
ECI: Hypothyroidism	11.18%	-0.25	0.02	<0.001	0.78	0.75–0.81
ECI: Hypertension, uncomplicated	42.94%	-0.5	0.02	<0.001	0.61	0.59–0.63
ECI: Lymphoma	2.65%	0.41	0.04	<0.001	1.51	1.38–1.64
ECI: Solid tumor without metastasis	13.37%	0.19	0.02	<0.001	1.2	1.15–1.26
ECI: Psychoses	1.16%	-0.32	0.06	<0.001	0.73	0.64–0.82
ECI: Peripheral vascular disorders	15.70%	0.22	0.02	<0.001	1.25	1.2–1.29
ECI: Valvular disease	12.83%	-0.08	0.02	<0.001	0.92	0.89–0.96
ECI: Pulmonary circulation disorders	7.07%	0.11	0.03	<0.001	1.12	1.07–1.18
**Interaction effects with septic shock**^**b**^						
Sepsis as primary diagnosis	39.63%					
Effect in severe sepsis		-0.78	0.02	<0.001	0.46	0.44–0.48
Effect in septic shock		-0.49	0.03	<0.001	0.61	0.58–0.64
Abdominal infection	21.08%					
Effect in severe sepsis		-0.07	0.02	0.001	0.93	0.9–0.97
Effect in septic shock		-0.25	0.03	<0.001	0.78	0.74–0.82
Infection of lower respiratory tract	48.08%					
Effect in severe sepsis		0.23	0.02	<0.001	1.25	1.21–1.3
Effect in septic shock		-0.08	0.02	<0.001	0.92	0.88–0.96
Urinary tract infection	30.48%					
Effect in severe sepsis		-0.48	0.02	<0.001	0.62	0.59–0.64
Effect in septic shock		-0.59	0.03	<0.001	0.55	0.52–0.58
ECI: Metastatic cancer	6.70%					
Effect in severe sepsis		0.55	0.04	<0.001	1.74	1.61–1.87
Effect in septic shock		0.33	0.06	<0.001	1.39	1.24–1.56
ECI: Other neurological disorders	15.05%					
Effect in severe sepsis		0.18	0.02	<0.001	1.2	1.15–1.25
Effect in septic shock		0.03	0.03	0.373	1.03	0.97–1.09
Leucemia	2.41%					
Effect in severe sepsis		0.41	0.06	<0.001	1.5	1.34–1.69
Effect in septic shock		0.86	0.09	<0.001	2.37	1.98–2.83
ECI: Obesity	9.79%					
Effect in severe sepsis		-0.23	0.03	<0.001	0.79	0.75–0.84
Effect in septic shock		-0.06	0.04	0.131	0.94	0.87–1.02
ECI: Paralysis	9.12%					
Effect in severe sepsis		-0.04	0.03	0.199	0.96	0.9–1.02
Effect in septic shock		-0.4	0.04	<0.001	0.67	0.62–0.73
Chemotherapy	4.02%					
Effect in severe sepsis		-0.32	0.06	<0.001	0.73	0.65–0.81
Effect in septic shock		0.17	0.11	0.126	1.18	0.95–1.46
Palliatve care	1.73%					
Effect in severe sepsis		0.94	0.08	<0.001	2.57	2.2–3
Effect in septic shock		0.14	0.12	0.249	1.15	0.9–1.47

Results based on 134,851cases with severe sepsis or septic shock treated in German hospitals in 2015. SD: Standard deviation, SE: Standard error, CI: confidence interval, CCI: Charlson Comorbidity Index, ECI: Elixhauser Comorbidity Index. Area under the curve is 0.741 (95% CI: 0.739, 0.744); *R*^*2*^ (squared Pearson correlation between hospital mortality and log-odds of mortality risk) is 0.17.

^a^ To allow for non-linear effects of age quadratic and cubic polynomials were added as predictors. Age was transformed so that estimate and odds ratio represent the effect per change of 10 years of age compared to age of 70.

^b^ All interaction effects were significant at α-level of 0.0003. To simplify the interpretation of the results we report the conditional effect estimates (effect given severe sepsis [no shock] vs. effect given septic shock).

**Fig 2:**
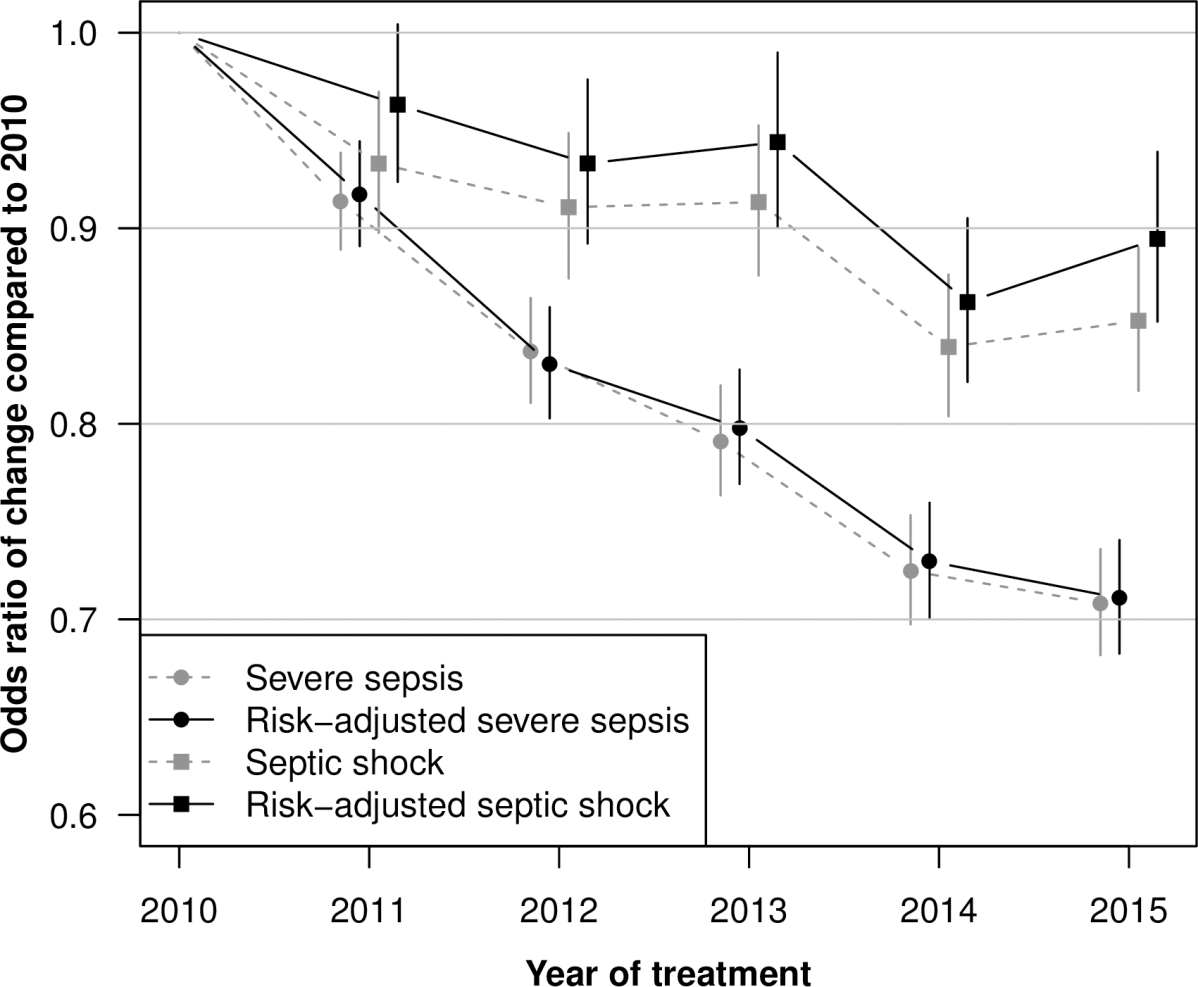
Secular trends for hospital mortality between 2010–2015 in cases with severe sepsis or septic shock. Dashed lines present odds ratios compared to 2010 without controlling for mortality risk-factors, solid lines present odds ratios compared to 2010 while controlling for risk-factors. Results based on a logistic regression using generalized estimating equations.

Fig 2 presents the secular trends between 2010 and 2015 in mortality for cases with severe sepsis and cases with septic shock. The decreasing trajectories remained significant also when controlled for differences in risk-factors.

## Discussion

This study developed a risk-model based on German national DRG data, which is suitable for comparing sepsis-related mortality between German hospitals. To our knowledge this is the first European claims-based risk-model for sepsis-related mortality. It is also the first risk-model using a full national sample of inpatient cases, securing validity of its use among German hospitals. The model showed good discrimination, calibration and fit, comparable to previous claims-based models for mortality in sepsis and several other conditions [16, 23, 33, 34, 41, 42]. Additionally, the stability of the model across a time-lag of two years was proven.

We identified risk-factors among predispositions and characteristics of the infection. Beside these, severity of acute illness is considered to be a major risk-factor for sepsis-mortality [25, 43, 44]. Two previous claims-based risk-models included measures of presenting acute severity of illness, like use of vasopressors, early ventilation, and ICU care at admission [16, 23]. However, we did not include such indicators because procedure codes in German DRG data have no reliable time-flag to identify use of medication or procedures closely following hospital admission. Later use of organ support or ICU treatment cannot strictly be classified as patient-related risk-factors and might contain treatment-related complications biasing the risk-adjustment [45]. Yet, discrimination of our risk-model was only slightly lower compared to these previous models. Additionally, risk-models based on clinical data and incorporating acute illness severity did not show substantially higher discrimination compared to our new model [25, 44].

We observed some paradoxical protective effects of comorbidities like drug abuse, depression or complicated hypotension. Similar protective effects were also reported in the study by Ford et al [16] as well as in the developmental study of the Elixhauser comorbidity index [30]. Elixhauser et al. [30] explain these paradoxical effects by a coding bias caused by increased coding of less severe comorbidities in patients with few severe comorbidities and lower risk of mortality. Additionally, in patients that die quickly there might also be less coding of less severe diseases.

Since there is a time-lag of 1.5 years in availability of the national German DRG data, we needed to proof the stability of the model over time. A model developed using data of 2013 did not decrease in predictive validity when applied to the cases of 2015. This indicates that a model based on two years old national DRG data is suitable to be used for risk-adjustment in recently treated cases.

A secular trend of decreasing sepsis-related mortality has been reported for several developed countries [37–39, 46]. These changes probably result from the so called Will Rogers phenomenon [47]. It states that decreased mortality might result from increased awareness for a disease and increased diagnosis of less severely ill cases. We could show that a trajectory of decreased mortality was also existent when an adjustment for risk-factors was done. This indicates that changes might not solely result from the Will Rogers phenomenon and that modest but true improvements in quality of sepsis care might have occurred in Germany.

Based on the identified risk-factors and their coefficients estimates, the standardized mortality ratio (“observed over expected ratio”) and risk standardized mortality rate along with their confidence limits can be obtained to measure provider performance in sepsis care [19, 48]. Additionally, a smoothed risk-adjusted rate can be calculated that incorporates a shrinkage factor which corrects for unreliability in estimates based on small case numbers [20]. Relying solely on readily available DRG data, risk-adjusted indicators can be obtained with minimal efforts. The developed risk-model is used for internal benchmark and public reporting of sepsis-related mortality of 75 hospitals participating in the German Quality Network Sepsis, a voluntary quality initiative founded in 2016. Likewise, every German hospital could use the risk-model to compare its performance to the German average. Additionally, the model can be used for risk-adjusted comparison of mortality between German counties or states. The risk-model is not suitable as a clinical score for predicting risk of death for individual patients, since it does not incorporate relevant clinical and laboratory variables.

This study has several limitations. Administrative data have certain shortcomings that might bias hospital comparisons, like payment-related incentives for coding, over or under-coding of conditions or risk-factors, and inconsistencies in coding between hospitals [11]. The problem of under-coding of cases with sepsis has been reported in several studies from the USA, Sweden, Canada, and Australia [49]. This was also shown in a recent pilot-study in a German university hospital [50]. It is currently unknown how this might affect risk-adjusted hospital comparisons. Since no appropriate German clinical data bases or registries for patients with sepsis exist, we could not validate our risk-model against a risk-model based on validated clinical data, as was done in previous studies [23, 33, 34, 41, 51]. Additional limitations are caused by specific shortcomings of German DRG data. Since patients are anonymized, related hospital admissions could not be identified. Additionally, only hospital mortality but no mortality measured after a given time period (e.g. 90-days) was available. Thus, differences in discharge strategies between hospitals might bias performance estimates [52, 53]. ICD-10 codes are not classified as present at admission, which leads to possible bias [45]. We focused on conditions that most likely occur as comorbidities by relying on established indices [29, 30]. Finally, using the model derived from two year old national DRG data on recently treated cases might result in underestimated–or less likely overestimated–risk-adjusted mortality because of unknown secular changes.

## Conclusions

We developed a risk model for mortality in severe sepsis and septic shock based on claims data. The model showed good predictive validity, comparable to previous claims-based models as well as to clinical risk scores. The model can be used as an external risk-adjustment algorithm for evaluating and comparing sepsis-related mortality in German hospitals with minimal effort and costs. The availability of the model can promote measurement and improvement of quality of sepsis care in Germany. Future studies should investigate the validity of coding of risk-factors in German claims data and compare the validity of a claims-based model against models derived from validated clinical data.

## Supporting information

S1 Appendix Power calculation to determine the exclusion criterion in backward selection of risk-factors. (DOCX)

S1 Fig CONSORT flow diagram. (DOCX)

S1 Table Definitions of candidate variables for the risk-model. (DOCX)

S2 Table Coefficients estimates of logistic regression model using generalized estimation equations for hospital mortality in cases with severe sepsis or septic shock treated in 2013. (DOCX)
