# Supplementary material for: A risk-model for hospital mortality among patients with severe sepsis or septic shock based on German national administrative claims data
Source: PLoS One. 2018 Mar 20;13(3):e0194371. doi: 10.1371/journal.pone.0194371 (PMC5860764; doi:10.1371/journal.pone.0194371)
Supplement: S1 Appendix — (DOCX) [file pone.0194371.s001.docx]

S1 Appendix. Power calculation to determine the exclusion criterion in backward selection of risk-factors.

Because of the large sample size we chose a more restrictive exclusion criterion than *p* > 0.05. This exclusion criterion was identified by a power analysis [1]. The analysis was done to identify a two tailed *α*-level to meet the following assumptions:

- identification of a single dichotomous predictor with effect of *OR*=1.1 and incidence of 10%
- in a sample of 114,000 cases with baseline mortality of 44%
- given an explained variance by other predictors of 15%
- aiming at a test power of 0.8

The analysis was done using the software G-Power, version 3.1 [2].

References

1. Demidenko E. Sample size determination for logistic regression revisited. Stat Med. 2007;26(18):3385-97. doi: 10.1002/sim.2771.

2. Faul F, Erdfelder E, Buchner A, Lang A-G. Statistical power analyses using G*Power 3.1: Tests for correlation and regression analyses. Behavior Research Methods. 2009;41(4):1149-60.
