# Supplementary figures and images for: A risk-model for hospital mortality among patients with severe sepsis or septic shock based on German national administrative claims data
Source: PLoS One. 2018 Mar 20;13(3):e0194371. doi: 10.1371/journal.pone.0194371 (PMC5860764; doi:10.1371/journal.pone.0194371)

S1 Fig. CONSORT flow diagram.

a. Derivation cohort (2013)


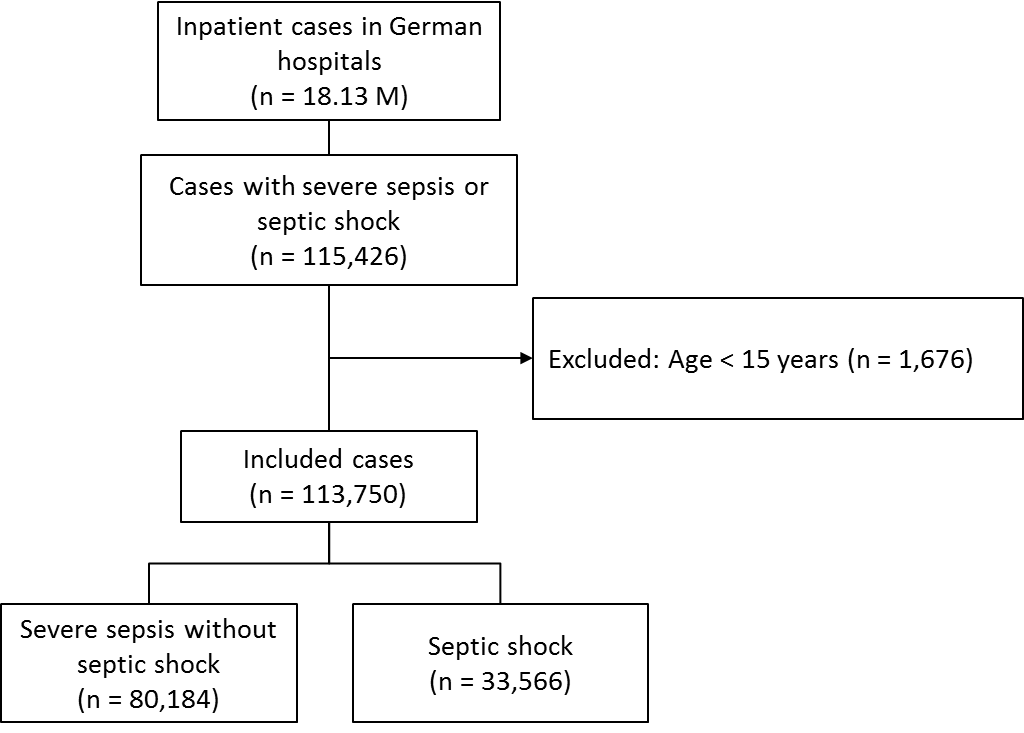


b. Validation cohort (2015)


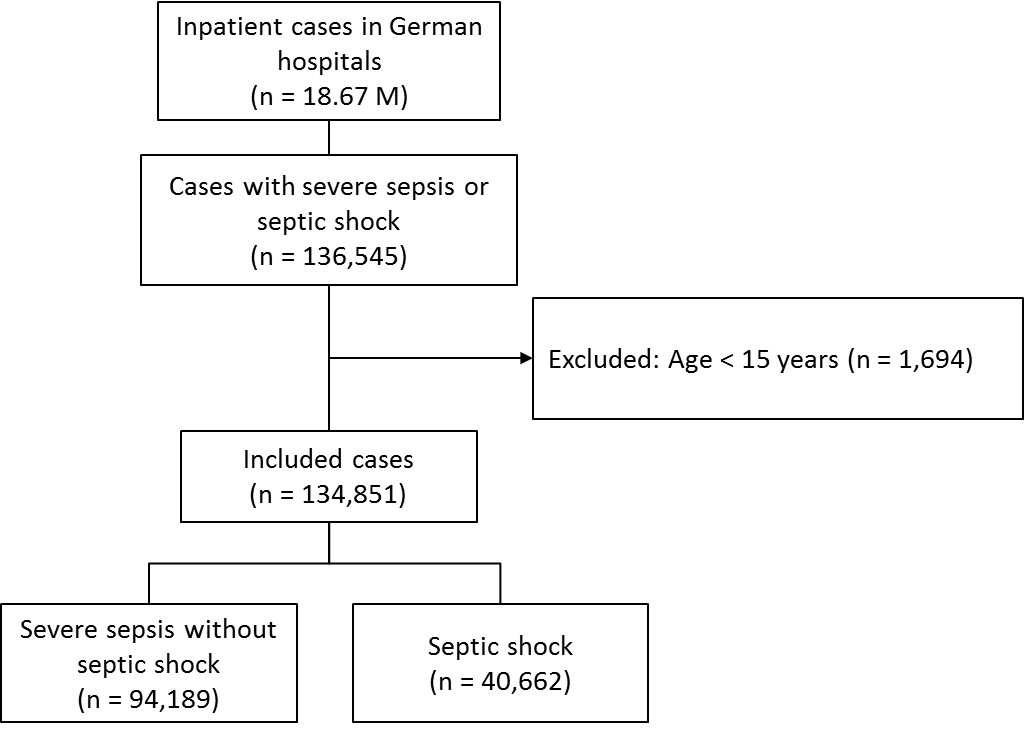

Supplement: S1 Fig — (DOCX) [file pone.0194371.s002.docx]
