# Supplementary material for: A risk-model for hospital mortality among patients with severe sepsis or septic shock based on German national administrative claims data
Source: PLoS One. 2018 Mar 20;13(3):e0194371. doi: 10.1371/journal.pone.0194371 (PMC5860764; doi:10.1371/journal.pone.0194371)
Supplement: S1 Table — (DOCX) [file pone.0194371.s003.docx]

**S1 Table.** **Definitions of candidate variables for the risk-model.**

| **Candidate variable** | **Definition** |
| --- | --- |
| **Patient demographics** |  |
| Gender | Coded "0" if male and "1" if female |
| Age in steps of 10 years centered at 70 years | age_t_ = (age - 70) / 10; |
| **Hospital admission** |  |
| Hospital admission type | Admission types in DRG data were collapsed because of small case numbers in types “Z” and “R”: 1. Emergency (type “N”); 2. Referral by physician or dentist (type “E” or “Z“); 3. Hospital transfer with pre-treatment >24h (type “V”); 4. Hospital transfer with pre-treatment < 24h or rehabilitation hospital (type “A” or “R”) |
| Admission to hospital by surgical department | Medical specialty code of first treating department: 1300; 1390; 1391; 1392; 1500; 1513; 1516; 1518; 1519; 1520; 1523; 1536; 1550; 1551; 1590; 1591; 1592; 1600; 1690; 1691; 1692; 1700; 1790; 1791; 1792; 1800; 1890; 1891; 1892; 1900; 1990; 1991; 1992; 2000; 2021; 2036; 2050; 2090; 2091; 2092; 2100; 2118; 2120; 2136; 2150; 2190; 2191; 2192; 2300; 2309; 2315; 2316; 2390; 2391; 2392; 3500; 3590; 3591; 3592; 3755; 3757 |
| **Clinical characteristics of the infection** |  |
| Septic shock | ICD-10 codes in primary or secondary diagnoses: R572 |
| Sepsis as primary diagnosis^a^ | ICD-10 codes in primary diagnosis: A400; A401; A402; A403; A408; A409; A410; A411; A412; A413; A414; A4151; A4152; A4158; A418; A419; R572; R650; R651 |
| Infection of lower respiratory tract | ICD-10 codes in primary or secondary diagnoses: J12; J13; J14; J15; J16; J17; J18; J20; J21; J22; J440; J441; J47; J86; J85; A15; A16; U6900 |
| Infection of upper respiratory tract | ICD-10 codes in primary or secondary diagnoses: J09; J10; J11; J00; J01; J02; J03; J04; J06; J05; A36; A37 |
| Abdominal infection | ICD-10 codes in primary or secondary diagnoses: A00; A01; A02; A03; A04; A05; A06; A07; A08; A09; K35; K37; K36; K5712; K5702; K5713; K5703; K5722; K5732; K5723; K5733; K5742; K5743; K5752; K5753; K5782; K5783; K5792; K5793; K61; K65; K67; K630; K631; K750; K751; K810 |
| Soft tissue and wound infections | ICD-10 codes in primary or secondary diagnoses: A46; B35; B36; B47; L03; L04; L08; L88; L05; B00 |
| Urinary tract infection | ICD-10 codes in primary or secondary diagnoses: N10; N11; N12; N151; N159; N16; N288; N34; N30; N390; N41; N45; N51; N482; N49; N70; N71; N72; N73; N74; N75; N76; N77; N61 |
| Infection of central nervous system | ICD-10 codes in primary or secondary diagnoses: A39; G00; G01; G02; G03; G04; G05; G06; G07; G08; A17 |
| Infection of vascular system | ICD-10 codes in primary or secondary diagnoses: I30; I32; I33; I39; I40; I41; I80 |
| Foreign body associated infection | ICD-10 codes in primary or secondary diagnoses: T826; T827; T835; T836; T845; T846; T847; T857 |
| Treatment of multiresistant pathogens | German procedure codes: 8987 |
| **Comorbidities^b^** |  |
| CCI: Myocardial infarction | ICD-10 codes in primary or secondary diagnoses: I211; I212; I229; I2522; I210; I219; I2529; I221; I213; I2521; I214; I220; I228; I2520 |
| CCI: Cerebrovascular disease | ICD-10 codes in primary or secondary diagnoses: G4589; G462; G4583; G4503; G4592; G4539; G4599; G460; G461; G4593; I600; G4542; G4543; G4523; G467; H340; I606; G4509; G4512; G4513; G4519; G4522; I612; I613; I601; I602; I603; I604; I605; G468; I607; I608; I609; I610; I611; I630; I631; G463; G464; G465; G466; I619; G4502; I639; I64; I650; I651; I652; I653; I658; I659; I660; I661; I662; I663; I664; I668; I669; I670; I6710; I6711; I672; I673; I674; I614; I615; I616; I618; I6788; I6200; I6201; I6202; I6209; I621; I629; I691; I692; I632; I633; I634; I635; I636; I638; I679; G4549; G4582; I682; I688; I690; G4533; I6780; G4529; G4532; I676; I681; I698; I680; I693; I677; I675; I694 |
| CCI: Dementia | ICD-10 codes in primary or secondary diagnoses: F012; F021; F011; F001; F03; F009; F010; F051; F013; F018; F019; F023; F000; F002; F022; F024; F028; G300; G301; G308; F020; G311; G309 |
| CCI: Peptic ulcer disease | ICD-10 codes in primary or secondary diagnoses: K270; K266; K267; K289; K271; K272; K269; K264; K265; K250; K251; K252; K253; K254; K255; K256; K257; K259; K260; K261; K262; K263; K280; K281; K282; K283; K284; K285; K286; K273; K274; K275; K276; K277; K279; K287 |
| CCI: Mild liver disease | ICD-10 codes in primary or secondary diagnoses: B180; B181; B182; B188; B189; K746; K760; K762; K763; K764; K702; K703; K739; K768; K769; K713; K714; K715; K717; K732; K738; K700; K701; Z944; K742; K743; K709; K745; K741; K744; K731; K730; K740 |
| CCI: Moderate or severe liver disease | ICD-10 codes in primary or secondary diagnoses: K704; K711; K765; K767; I982; K729; K766; I864; I850; I859; I983; K721 |
| ECI: Congestive heart failure | ICD-10 codes in primary or secondary diagnoses: I4288; I4280; I5019; I427; I509; I432; I438; I430; I431; P290; I099; I5012; I5013; I5014; I420; I425; I426; I5011; I5001; I429; I5000; I255 |
| ECI: Cardiac arrhythmias | ICD-10 codes in primary or secondary diagnoses: I459; I495; I498; I442; I471; I443; I456; I492; I489; I494; Z950; I483; I470; I479; R001; R008; I499; I441; Z4508; I480; I490; I493; R000; Z4500; I481; I472; I491; Z450; Z4501; I484; I482; T821; Z4502 |
| ECI: Valvular disease | ICD-10 codes in primary or secondary diagnoses: A520; I3480; I3488; I349; I350; I351; Z952; Z953; Z954; I360; I361; I340; I341; I342; I370; I371; I372; I378; I379; I352; I358; I359; I392; I393; I362; I368; I369; I051; I052; I058; I059; Q230; I38; I390; I391; I069; I070; I394; I398; I050; I079; I080; I081; I082; I083; Q231; Q232; Q233; I098; I068; I071; I072; I078; I062; I088; I089; I091; I061; I060 |
| ECI: Pulmonary circulation disorders | ICD-10 codes in primary or secondary diagnoses: I288; I2720; I2728; I280; I278; I260; I269; I271; I270; I289; I279 |
| ECI: Peripheral vascular disorders | ICD-10 codes in primary or secondary diagnoses: I7021; I7020; I7103; I7104; I738; I7023; I7102; I731; I792; I739; I771; I790; I709; I7100; I7101; I719; I701; I700; I7024; I7105; I7022; I7107; Z9580; I7025; I708; I7029; I714; Z9581; Z9588; Z959; K559; I711; I712; I7106; I713; I715; I716; I7026; K5588; K551; K5581; I718; K5582 |
| ECI: Hypertension, uncomplicated | ICD-10 codes in primary or secondary diagnoses: I1010; I1091; I1011; I1001; I1090; I1000 |
| ECI: Hypertension, complicated | ICD-10 codes in primary or secondary diagnoses: I1101; I1101; I1191; I1200; I1320; I1310; I1100; I1501; I1301; I1320; I1310; I1190; I1311; I1300; I1321; I1301; I1100; I1510; I1511; I1520; I1311; I1580; I1390; I1200; I1290; I1291; I1201; I1300; I1500; I1521; I1591; I1581; I1201; I1321; I1391; I1590 |
| ECI: Paralysis | ICD-10 codes in primary or secondary diagnoses: G8212; G8266; G833; G8211; G8263; G8264; G8267; G831; G832; G8265; G8201; G8341; G8349; G8200; G041; G8261; G8233; G8239; G8240; G8213; G8219; G819; G830; G811; G8340; G8203; G8209; G839; G8210; G8260; G8262; G8221; G8241; G8242; G114; G8229; G8222; G8250; G8202; G8220; G8230; G8232; G802; G8223; G8269; G810; G8231; G8259; G8252; G8253; G8249; G801; G8251; G8243 |
| ECI: Other neurological disorders | ICD-10 codes in primary or secondary diagnoses: G130; G934; G328; G350; G112; G131; G129; G931; G369; G319; G138; G121; G122; G132; G128; G210; G211; G119; G213; G214; G2000; G212; G118; G3521; G120; G3531; G359; G2091; G3188; G320; G10; G110; G111; G113; G2001; G2010; G3520; G375; G378; G379; G3530; G4001; G4002; G360; G361; G370; G371; G372; G373; G374; G406; G407; G408; G4000; G410; G411; G4008; G4009; G401; G402; G403; G404; G405; G218; G219; G22; G409; G2020; G2021; G3182; G412; G418; G419; R470; G3510; G3511; G114; G312; G2090; G2011; G255; G368; R560; R568; G3181; G254 |
| ECI: Chronic pulmonary disease | ICD-10 codes in primary or secondary diagnoses: J418; I279; J679; J42; J678; J431; J670; J671; J672; I278; J411; J4411; J4412; J4413; J4419; J432; J684; J661; J668; J4401; J4402; J4403; J4409; J4410; J4499; J450; J451; J458; J4480; J4481; J4482; J662; J4489; J4490; J4491; J4492; J4493; J632; J633; J634; J430; J638; J64; J438; J4483; J4400; J61; J701; J703; J631; J40; J410; J673; J674; J675; J676; J677; J439; J60; J630; J620; J628; J65; J635; J459; J46; J660; J47 |
| ECI: Diabetes, uncomplicated | ICD-10 codes in primary or secondary diagnoses: E1201; E1091; E1490; E1011; E1001; E1390; E1290; E1090; E1491; E1411; E1301; E1111; E1391; E1211; E1191; E1311; E1291; E1401; E1101; E1190 |
| ECI: Diabetes, complicated | ICD-10 codes in primary or secondary diagnoses: E1040; E1041; E1031; E1241; E1050; E1441; E1231; E1240; E1072; E1261; E1030; E1230; E1021; E1081; E1051; E1060; E1080; E1061; E1431; E1221; E1251; E1273; E1381; E1250; E1075; E1151; E1160; E1220; E1440; E1173; E1260; E1161; E1272; E1461; E1131; E1451; E1074; E1150; E1421; E1020; E1430; E1172; E1361; E1450; E1175; E1460; E1130; E1321; E1140; E1141; E1420; E1274; E1275; E1280; E1360; E1281; E1174; E1373; E1180; E1181; E1380; E1330; E1073; E1474; E1341; E1350; E1351; E1481; E1375; E1372; E1320; E1374; E1340; E1121; E1475; E1331; E1473; E1480; E1120; E1472 |
| ECI: Hypothyroidism | ICD-10 codes in primary or secondary diagnoses: E012; E018; E011; E033; E010; E030; E000; E038; E02; E039; E009; E031; E032; E890; E001; E002; E035; E034 |
| ECI: Renal failure | ICD-10 codes in primary or secondary diagnoses: N250; Z490; Z491; Z492; N19; Z940; N181; N182; N183; N184; N185; N1880; N1889; N189; Z992 |
| ECI: Lymphoma | ICD-10 codes in primary or secondary diagnoses: C845; C846; C848; C810; C849; C851; C847; C857; C817; C859; C852; C965; C966; C967; C968; C969; C826; C827; C829; C830; C831; C9030; C819; C820; C821; C822; C823; C824; C825; C864; C865; C811; C812; C813; C814; C9031; C960; C962; C964; C838; C841; C844; C8890; C8891; C9000; C9001; C9020; C9021; C8821; C8830; C8831; C860; C861; C862; C863; C840; C8801; C8820; C866; C8800; C8840; C8841; C8870; C833; C839; C835; C837; C8871 |
| ECI: Metastatic cancer | ICD-10 codes in primary or secondary diagnoses: C793; C797; C794; C7983; C788; C791; C792; C774; C775; C795; C790; C796; C783; C778; C779; C7981; C770; C771; C772; C773; C799; C800; C809; C784; C780; C781; C782; C7984; C785; C786; C787; C7982; C7988 |
| ECI: Solid tumor without metastasis | ICD-10 codes in primary or secondary diagnoses: C07; C080; C068; C069; C161; C154; C155; C158; C159; C160; C248; C081; C088; C089; C090; C166; C091; C169; C001; C002; C003; C004; C005; C006; C162; C163; C164; C165; C021; C022; C023; C024; C028; C029; C030; C031; C039; C040; C041; C048; C049; C221; C222; C223; C224; C227; C229; C061; C062; C383; C384; C388; C390; C398; C399; C400; C168; C000; C098; C099; C100; C101; C102; C103; C008; C009; C01; C020; C111; C112; C113; C118; C119; C12; C130; C131; C132; C138; C139; C140; C142; C148; C150; C151; C152; C153; C460; C461; C462; C463; C467; C468; C469; C470; C471; C472; C401; C402; C475; C170; C171; C172; C173; C178; C104; C108; C109; C110; C183; C184; C185; C186; C187; C188; C189; C19; C20; C210; C211; C212; C218; C220; C508; C509; C510; C511; C512; C518; C519; C52; C530; C531; C538; C539; C540; C541; C473; C474; C548; C549; C55; C56; C570; C571; C572; C573; C574; C577; C578; C579; C58; C600; C601; C602; C608; C609; C61; C620; C621; C629; C630; C631; C632; C637; C638; C639; C64; C65; C66; C670; C671; C672; C673; C674; C675; C676; C677; C678; C679; C403; C408; C409; C4101; C4102; C411; C412; C4130; C4131; C4132; C414; C418; C419; C430; C431; C432; C433; C434; C435; C436; C437; C438; C439; C450; C451; C452; C457; C459; C722; C723; C724; C725; C728; C729; C73; C740; C741; C749; C750; C751; C752; C753; C754; C755; C758; C759; C760; C761; C762; C763; C764; C765; C767; C768; C495; C496; C498; C499; C500; C501; C502; C503; C504; C505; C506; C050; C051; C052; C058; C059; C060; C23; C240; C241; C269; C249; C250; C251; C542; C543; C254; C257; C258; C259; C260; C261; C268; C328; C300; C301; C310; C311; C312; C313; C318; C319; C320; C321; C322; C323; C690; C329; C33; C340; C341; C717; C718; C719; C720; C721; C37; C381; C382; C711; C691; C97; C693; C694; C695; C696; C252; C253; C680; C681; C688; C689; C479; C712; C692; C714; C715; C716; C182; C698; C699; C700; C701; C709; C710; C180; C480; C713; C179; C343; C181; C488; C342; C380; C348; C349; C476; C492; C493; C482; C481; C478; C494; C490; C491 |
| ECI: Rheumatoid arthritis/collagen vascular diseases | ICD-10 codes in primary or secondary diagnoses: L941; L940; M0511; L943; M0501; M0510; M0537; M0538; M0539; M0500; M0581; M0502; M0503; M0504; M0505; M0506; M0507; M0508; M0509; M0590; M0591; M0512; M0513; M0514; M0515; M0516; M0517; M0518; M0519; M0520; M0521; M0522; M0523; M0524; M0525; M0526; M0527; M0528; M0529; M0530; M0531; M0532; M0533; M0534; M0535; M0536; M0617; M0618; M0619; M0580; M0621; M0582; M0583; M0584; M0585; M0586; M0587; M0588; M0589; M0630; M0631; M0632; M0633; M0634; M0635; M0636; M0637; M0638; M0639; M0640; M0641; M0642; M0643; M0644; M0645; M0646; M0647; M0648; M0649; M0680; M0681; M0682; M0683; M0684; M0685; M0686; M0687; M0688; M0689; M0690; M0691; M0692; M0693; M0694; M0695; M0696; M0697; M0698; M0699; M0800; M0801; M0802; M0803; M0804; M0805; M0806; M0807; M0808; M0809; M0810; M0811; M0812; M0813; M0814; M0815; M0816; M0817; M0818; M0819; M0820; M0821; M0822; M0823; M0824; M0825; M0826; M0827; M0828; M0829; M083; M0840; M0841; M0842; M0843; M0844; M0845; M0846; M0847; M0848; M0849; M0870; M0871; M0592; M0593; M0594; M0595; M0596; M0597; M0598; M0599; M0600; M0601; M0602; M0603; M0604; M0605; M0606; M0607; M0608; M0609; M0610; M0611; M0612; M0613; M0614; M0615; M0616; M0897; M0898; M0899; M0620; M1201; M0622; M0623; M0624; M0625; M0626; M0627; M0628; M0629; M1230; M1231; M1232; M1233; M1234; M1235; M1236; M1237; M1238; M1239; M300; M301; M302; M303; M308; M310; M311; M312; M313; M320; M321; M328; M329; M330; M331; M332; M339; M340; M341; M342; M348; M349; M350; M351; M352; M353; M354; M355; M356; M357; M358; M359; M4500; M4501; M4502; M4503; M4504; M4505; M4506; M4507; M4508; M4509; M461; M4680; M4681; M4682; M4683; M4684; M4685; M4686; M4687; M4688; M4689; M4690; M4691; M4692; M4693; M4694; M4695; M4696; M4697; M4698; M4699; M1202; M1203; M1204; M1205; M1206; M1207; M1208; M1209; M0883; M0884; M0872; M0873; M0874; M0875; M0876; M0877; M0878; M0879; M0880; M0881; M0882; M0896; M0888; M0885; M0886; M0887; M0892; M0889; M0890; M0891; M1200; M0893; M0894; M0895 |
| ECI: Coagulopathy | ICD-10 codes in primary or secondary diagnoses: D6835; D686; D681; D6838; D6834; D691; D6832; D684; D685; D67; D680; D6952; D6833; D6821; D6824; D6825; D6820; D650; D651; D652; D659; D66; D6940; D6941; D6823; D6953; D6957; D6822; D6831; D6959; D6826; D6828; D6961; D693; D693; D6960; D6958; D689; D688 |
| ECI: Obesity | ICD-10 codes in primary or secondary diagnoses: E6699; E6629; E6602; E6622; E6692; E6680; E6609; E6691; E6621; E6611; E6601; E6690; E6689; E6681; E6612; E6610; E6620; E6600; E6682; E6619 |
| ECI: Fluid and electrolyte disorders | ICD-10 codes in primary or secondary diagnoses: E875; E222; E874; E876; E877; E86; E871; E872; E873; E878; E870 |
| ECI: Blood loss anemia | ICD-10 codes in primary or secondary diagnoses: D500 |
| ECI: Deficiency anemia | ICD-10 codes in primary or secondary diagnoses: D508; D512; D509; D520; D521; D528; D538; D510; D511; D529; D518; D513; D539; D532; D530; D519; D531 |
| ECI: Alcohol abuse | ICD-10 codes in primary or secondary diagnoses: F108; K703; F104; T510; I426; K700; G621; F105; T518; T511; Z502; F109; T513; K709; F103; T512; F102; E52; F107; K292; F100; T519; F101; F106 |
| ECI: Drug abuse | ICD-10 codes in primary or secondary diagnoses: F128; F122; F121; F134; F127; F133; F126; F131; F166; F167; F130; F160; F161; F162; F159; F125; F165; F138; F139; F129; F169; F180; F132; F144; F181; F182; F184; F135; F136; F123; F110; F111; F112; F113; F168; F115; F116; F145; F146; F147; F148; F149; F137; F164; F188; F189; F190; F114; F192; F193; F117; F118; F119; F120; F185; F124; F187; F150; F151; F152; F191; F154; F155; F194; F195; F196; F183; F198; F186; F163; F140; F141; F156; F153; F157; F197; F158; F143; F199; F142 |
| ECI: Psychoses | ICD-10 codes in primary or secondary diagnoses: F24; F239; F220; F238; F232; F230; F233; F251; F252; F202; F203; F259; F208; F209; F28; F200; F231; F312; F228; F258; F250; F204; F205; F315; F29; F302; F201; F229; F206 |
| ECI: Depression | ICD-10 codes in primary or secondary diagnoses: F323; F328; F314; F322; F204; F315; F329; F333; F321; F432; F313; F332; F330; F331; F338; F339; F320; F334; F412; F341 |
| Leucemia | ICD-10 codes in primary or secondary diagnoses: C901; C91; C92; C93; C94; C95 |
| **Specific procedures** |  |
| Palliatve care | German procedure codes: 8982; 898e |
| Stroke treatment | German procedure codes: 8981; 898b |
| Chemotherapy | German procedure codes: 854 |
| **Candidate variables excluded because of incidence < 1%** |  |
| Congenital asplenia (incidence 0.02%) | Q890 |
| Pregnancy-associated infections (incidence: 0.04%) | O753; O85; O03; O04; O05; O06; O07; O080 |
| Acquired asplenia (incidence: 0.14%) | D730 |
| ECI: HIV (incidence: 0.23%) | B20; B22; B21; B24 |

CCI: Charlson Comorbidity Index. ECI: Elixhauser Comorbidity Index. Variables based on ICD-10 codes, procedure codes, or medical specialty codes were given the value “1” if one of the specified codes was present, else they were given the value “0”. The string-matching of codes was based on the comparison of the 1:*n* first characters, were *n* is the number of characters in the codes specified in the table (e.g. string-matching for J12 identified all cases with diagnoses J12.0, J12.1, J12.2, J12.3, J.12.8, and J12.9).

^a^ Sepsis was identified by clinical and pathogen based sepsis codes. The selection of codes resembles the definition used for sepsis in the currently largest claims-based quality initiative in Germany (<https://www.initiative-qualitaetsmedizin.de/>).

^b^ Categories of the CCI, which had the same definition as corresponding categories of the ECI were excluded. If categories of CCI and ECI were not exactly identical but showed strong overlap, the category that had the stronger univariate relation to hospital mortality was used.
