# Supplementary material for: A risk-model for hospital mortality among patients with severe sepsis or septic shock based on German national administrative claims data
Source: PLoS One. 2018 Mar 20;13(3):e0194371. doi: 10.1371/journal.pone.0194371 (PMC5860764; doi:10.1371/journal.pone.0194371)
Supplement: S2 Table — (DOCX) [file pone.0194371.s004.docx]

**S2 Table. Coefficients estimates of logistic regression model using generalized estimation equations for hospital mortality in cases with severe sepsis or septic shock treated in 2013.**

| **Variable** | **Estimate** | **SE** | **P-value** | **Odds Ratio** | **95% CI** |
| --- | --- | --- | --- | --- | --- |
| Intercept | -0.25 | 0.03 | <0.001 |  |  |
| Female gender | 0.15 | 0.01 | <0.001 | 1.17 | 1.13-1.2 |
| Age (estimate based on transformed value)^a^ | 0.39 | 0.01 | <0.001 | 1.48 | 1.46-1.51 |
| Age^2^ | 0.04 | 0 | <0.001 | 1.04 | 1.03-1.05 |
| Age^3^ | 0.01 | 0 | <0.001 | 1.01 | 1-1.01 |
| Reason for admission: Emergency (reference) | 0 |  |  | 1 |  |
| Admission by physician | -0.09 | 0.02 | <0.001 | 0.92 | 0.88-0.95 |
| Hospital transfer with pre-treatment >24h | 0.18 | 0.03 | <0.001 | 1.2 | 1.14-1.26 |
| Hospital transfer with pre-treatment < 24h or rehabilitation hospital | -0.05 | 0.05 | 0.304 | 0.95 | 0.87-1.04 |
| Septic shock | 1.16 | 0.04 | <0.001 | 3.2 | 2.95-3.47 |
| Infection of central nervous system | -0.25 | 0.06 | <0.001 | 0.78 | 0.7-0.88 |
| Foreign body associated infection | -0.4 | 0.03 | <0.001 | 0.67 | 0.64-0.7 |
| Infection of vascular system | -0.23 | 0.03 | <0.001 | 0.79 | 0.74-0.85 |
| Infection of upper respiratory tract | -0.22 | 0.07 | 0.001 | 0.8 | 0.71-0.91 |
| Soft tissue and wound infections | -0.32 | 0.03 | <0.001 | 0.72 | 0.69-0.76 |
| CCI: Cerebrovascular disease | 0.13 | 0.02 | <0.001 | 1.13 | 1.08-1.19 |
| CCI: Myocardial infarction | 0.1 | 0.02 | 0.0001 | 1.1 | 1.05-1.16 |
| CCI: Mild liver disease | 0.24 | 0.03 | <0.001 | 1.27 | 1.2-1.34 |
| CCI: Moderate or severe liver disease | 0.95 | 0.04 | <0.001 | 2.58 | 2.37-2.81 |
| ECI: Blood loss anemia | -0.27 | 0.07 | <0.001 | 0.76 | 0.67-0.87 |
| ECI: Cardiac arrhythmias | 0.07 | 0.02 | <0.001 | 1.07 | 1.04-1.1 |
| ECI: Coagulopathy | 0.38 | 0.02 | <0.001 | 1.46 | 1.4-1.52 |
| ECI: Deficiency anemia | -0.35 | 0.03 | <0.001 | 0.71 | 0.66-0.75 |
| ECI: Depression | -0.58 | 0.03 | <0.001 | 0.56 | 0.53-0.59 |
| ECI: Drug abuse | -0.67 | 0.06 | <0.001 | 0.51 | 0.45-0.58 |
| ECI: Fluid and electrolyte disorders | -0.09 | 0.02 | <0.001 | 0.91 | 0.88-0.94 |
| ECI: Hypertension, complicated | -0.51 | 0.03 | <0.001 | 0.6 | 0.57-0.63 |
| ECI: Hypothyroidism | -0.3 | 0.02 | <0.001 | 0.74 | 0.71-0.78 |
| ECI: Hypertension, uncomplicated | -0.46 | 0.02 | <0.001 | 0.63 | 0.61-0.65 |
| ECI: Lymphoma | 0.43 | 0.05 | <0.001 | 1.54 | 1.41-1.68 |
| ECI: Metastatic cancer | 0.5 | 0.04 | <0.001 | 1.65 | 1.53-1.79 |
| Leucemia | 0.67 | 0.06 | <0.001 | 1.95 | 1.74-2.19 |
| ECI: Pulmonary circulation disorders | 0.12 | 0.03 | <0.001 | 1.13 | 1.07-1.19 |
| ECI: Psychoses | -0.27 | 0.07 | <0.001 | 0.76 | 0.67-0.87 |
| ECI: Peripheral vascular disorders | 0.26 | 0.02 | <0.001 | 1.3 | 1.26-1.35 |
| **Interaction effects with septic shock^b^** |  |  |  |  |  |
| Admission by a surgical department |  |  |  |  |  |
| Effect in severe sepsis | -0.03 | 0.03 | 0.327 | 0.98 | 0.93-1.03 |
| Effect in septic shock | -0.23 | 0.04 | <0.001 | 0.79 | 0.74-0.85 |
| Sepsis as primary diagnosis |  |  |  |  |  |
| Effect in severe sepsis | -0.7 | 0.02 | <0.001 | 0.49 | 0.47-0.52 |
| Effect in septic shock | -0.49 | 0.03 | <0.001 | 0.62 | 0.58-0.65 |
| Abdominal infection |  |  |  |  |  |
| Effect in severe sepsis | -0.07 | 0.02 | 0.001 | 0.93 | 0.9-0.97 |
| Effect in septic shock | -0.3 | 0.03 | <0.001 | 0.74 | 0.69-0.79 |
| Infection of lower respiratory tract |  |  |  |  |  |
| Effect in severe sepsis | 0.21 | 0.02 | <0.001 | 1.23 | 1.19-1.27 |
| Effect in septic shock | -0.11 | 0.03 | <0.001 | 0.9 | 0.85-0.95 |
| Urinary tract infection |  |  |  |  |  |
| Effect in severe sepsis | -0.49 | 0.02 | <0.001 | 0.61 | 0.59-0.63 |
| Effect in septic shock | -0.64 | 0.03 | <0.001 | 0.53 | 0.5-0.56 |
| ECI: Congestive heart failure |  |  |  |  |  |
| Effect in severe sepsis | 0.22 | 0.02 | <0.001 | 1.25 | 1.2-1.3 |
| Effect in septic shock | 0.06 | 0.03 | 0.028 | 1.06 | 1.01-1.12 |
| ECI: Solid tumor without metastasis |  |  |  |  |  |
| Effect in severe sepsis | 0.25 | 0.03 | <0.001 | 1.29 | 1.21-1.36 |
| Effect in septic shock | 0.04 | 0.04 | 0.286 | 1.04 | 0.97-1.12 |
| ECI: Other neurological disorders |  |  |  |  |  |
| Effect in severe sepsis | 0.2 | 0.02 | <0.001 | 1.22 | 1.17-1.29 |
| Effect in septic shock | -0.05 | 0.04 | 0.148 | 0.95 | 0.88-1.02 |
| ECI: Obesity |  |  |  |  |  |
| Effect in severe sepsis | -0.2 | 0.03 | <0.001 | 0.82 | 0.77-0.87 |
| Effect in septic shock | -0.02 | 0.04 | 0.568 | 0.98 | 0.9-1.06 |
| ECI: Paralysis |  |  |  |  |  |
| Effect in severe sepsis | -0.02 | 0.03 | 0.529 | 0.98 | 0.92-1.04 |
| Effect in septic shock | -0.3 | 0.05 | <0.001 | 0.74 | 0.67-0.82 |
| ECI: Renal failure |  |  |  |  |  |
| Effect in severe sepsis | -0.08 | 0.02 | <0.001 | 0.92 | 0.89-0.95 |
| Effect in septic shock | 0.09 | 0.03 | 0.004 | 1.09 | 1.03-1.16 |
| Chemotherapy |  |  |  |  |  |
| Effect in severe sepsis | -0.33 | 0.06 | <0.001 | 0.72 | 0.64-0.81 |
| Effect in septic shock | 0.15 | 0.09 | 0.12 | 1.16 | 0.96-1.39 |
| Palliative care |  |  |  |  |  |
| Effect in severe sepsis | 0.86 | 0.08 | <0.001 | 2.37 | 2.01-2.8 |
| Effect in septic shock | 0.05 | 0.14 | 0.726 | 1.05 | 0.8-1.38 |

Results based on 113,750 cases with severe sepsis or septic shock treated in German hospitals in 2013. SD: Standard deviation, SE: Standard error, CI: confidence interval, CCI: Charlson Comorbidity Index, ECI: Elixhauser Comorbidity Index. Area under the curve is 0.737 (95% CI; 0.734, 0.74); *R^2^* (squared Pearson correlation between hospital mortality and log-odds of mortality risk) is 0.16.

^a^ To allow for non-linear effects of age quadratic and cubic polynomials were added as predictors. Age was transformed so that estimate and odds ratio represent the effect per change of 10 years of age compared to age of 70.

^b^ All interaction effects were significant at α-level of 0.0003. To simplify the interpretation of the results we report the conditional effect estimates (effect given severe sepsis [no shock] vs. effect given septic shock).
